# Transmission from vaccinated individuals in a large SARS-CoV-2 Delta variant outbreak

**DOI:** 10.1016/j.cell.2021.12.027

**Published:** 2022-02-03

**Authors:** Katherine J. Siddle, Lydia A. Krasilnikova, Gage K. Moreno, Stephen F. Schaffner, Johanna Vostok, Nicholas A. Fitzgerald, Jacob E. Lemieux, Nikolaos Barkas, Christine Loreth, Ivan Specht, Christopher H. Tomkins-Tinch, Jillian S. Paull, Beau Schaeffer, Bradford P. Taylor, Bryn Loftness, Hillary Johnson, Petra L. Schubert, Hanna M. Shephard, Matthew Doucette, Timelia Fink, Andrew S. Lang, Stephanie Baez, John Beauchamp, Scott Hennigan, Erika Buzby, Stephanie Ash, Jessica Brown, Selina Clancy, Seana Cofsky, Luc Gagne, Joshua Hall, Rachel Harrington, Gabrielle L. Gionet, Katherine C. DeRuff, Megan E. Vodzak, Gordon C. Adams, Sabrina T. Dobbins, Sarah D. Slack, Steven K. Reilly, Lisa M. Anderson, Michelle C. Cipicchio, Matthew T. DeFelice, Jonna L. Grimsby, Scott E. Anderson, Brendan S. Blumenstiel, James C. Meldrim, Heather M. Rooke, Gina Vicente, Natasha L. Smith, Katelyn S. Messer, Faye L. Reagan, Zoe M. Mandese, Matthew D. Lee, Marianne C. Ray, Marissa E. Fisher, Maesha A. Ulcena, Corey M. Nolet, Sean E. English, Katie L. Larkin, Kyle Vernest, Sushma Chaluvadi, Deirdre Arvidson, Maurice Melchiono, Theresa Covell, Vaira Harik, Taylor Brock-Fisher, Molly Dunn, Amanda Kearns, William P. Hanage, Clare Bernard, Anthony Philippakis, Niall J. Lennon, Stacey B. Gabriel, Glen R. Gallagher, Sandra Smole, Lawrence C. Madoff, Catherine M. Brown, Daniel J. Park, Bronwyn L. MacInnis, Pardis C. Sabeti

**Affiliations:** 1 Broad Institute of Harvard and MIT, Cambridge, MA 02142, USA; 2 Department of Organismic and Evolutionary Biology, Harvard University, Cambridge, MA 02138, USA; 3 Department of Immunology and Infectious Diseases, Harvard T.H. Chan School of Public Health, Harvard University, Boston, MA 02115, USA; 4 Massachusetts Department of Public Health, Boston, MA 02199, USA; 5 Division of Infectious Diseases, Massachusetts General Hospital, Boston, MA 02114, USA; 6 Faculty of Arts and Sciences, Harvard University, Cambridge, MA 02138, USA; 7 Department of Systems Biology, Harvard Medical School, Boston, MA 02115, USA; 8 Department of Epidemiology, Harvard T.H. Chan School of Public Health, Harvard University, Boston, MA 02115, USA; 9 Center for Communicable Disease Dynamics, Department of Epidemiology, Harvard T. H. Chan School of Public Health, Harvard University, Boston, MA 02115, USA; 10 Applied Epidemiology Fellowship, Council of State and Territorial Epidemiologists, Atlanta, GA 30345, USA; 11 Barnstable County Department of Health and the Environment, Barnstable, MA 02630, USA; 12 Barnstable County Department of Human Services, Barnstable, MA 02630, USA; 13 Community Tracing Collaborative, Commonwealth of Massachusetts, Boston, MA 02199, USA; 14 Howard Hughes Medical Institute, Chevy Chase, MD 20815, USA; 15 Massachusetts Consortium for Pathogen Readiness, Boston, MA 02115, USA

**Keywords:** SARS-CoV-2, Delta, genomics, contact tracing, transmission, vaccination

## Abstract

An outbreak of over 1,000 COVID-19 cases in Provincetown, Massachusetts (MA), in July 2021—the first large outbreak mostly in vaccinated individuals in the US—prompted a comprehensive public health response, motivating changes to national masking recommendations and raising questions about infection and transmission among vaccinated individuals. To address these questions, we combined viral genomic and epidemiological data from 467 individuals, including 40% of outbreak-associated cases. The Delta variant accounted for 99% of cases in this dataset; it was introduced from at least 40 sources, but 83% of cases derived from a single source, likely through transmission across multiple settings over a short time rather than a single event. Genomic and epidemiological data supported multiple transmissions of Delta from and between fully vaccinated individuals. However, despite its magnitude, the outbreak had limited onward impact in MA and the US overall, likely due to high vaccination rates and a robust public health response.

## Highlights

• Large Delta outbreak in a highly vaccinated population changed US indoor masking policy
• Outbreak included multiple Delta introductions; one introduction led to 83% of cases
• Spread from and between vaccinated cases contributed, a picture resolved by genomic data
• Outbreak was successfully contained; it did not drive the rise of Delta in MA or the US

## Graphical abstract

**Figure:**
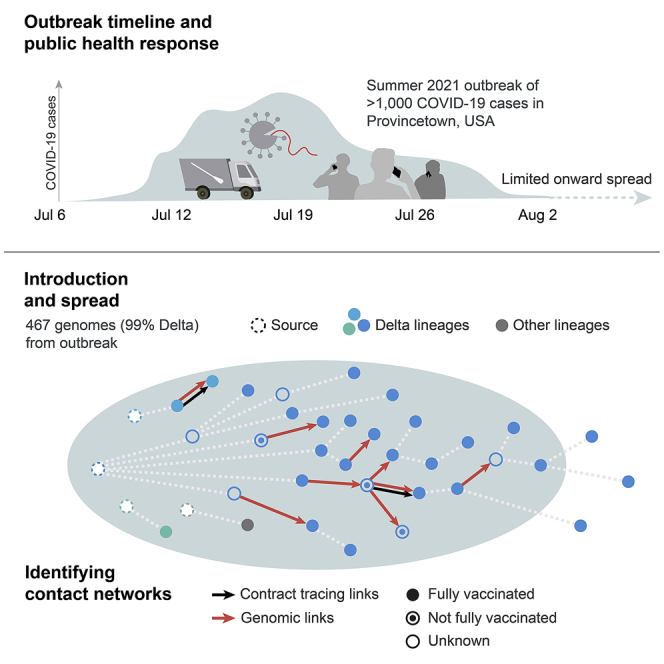

## Teaser

Rapid integration of epidemiological and genomic data was used to support the public health response during a large outbreak of the SARS-CoV-2 Delta variant. The outbreak stemmed primarily from a single source spreading the virus at multiple locations, and while numerous likely transmissions from and between vaccinated individuals were observed, the downstream impact of the outbreak was minimized with robust intervention and community engagement.

## Introduction

On July 10th, 2021, the Massachusetts Department of Public Health (MADPH) received reports of an increase in COVID-19 cases among people who resided in or had recently visited Provincetown, a tourist town on Cape Cod, MA. Provincetown had attracted thousands of visitors, many of whom reported attending multiple large public and private gatherings—often indoors and unmasked, in accordance with public health guidance at the time—starting with the 4th of July weekend and throughout the following week. COVID-19 incidence in the area rose quickly during the subsequent two-week period, from 0 cases in the 14 days before July 3rd to a peak of 456 cases per 100,000 persons per day during July 11th–24th.

Notably, 74% of the reported cases were fully vaccinated individuals, of whom 79% were symptomatic (Brown et al., 2021). As of July 1st, estimated COVID-19 full vaccination coverage among the eligible population was 68% in Barnstable County (where Provincetown is located) and 67% across MA (Massachusetts Department of Public Health, 2021). This was the first large, well-characterized outbreak of COVID-19 in a highly vaccinated population in the US and contributed to the CDC’s decision to reinstate their indoor mask recommendation for vaccinated individuals (CDC, 2021).

The rapid increase in cases despite high vaccination rates prompted state and local public health departments to launch a comprehensive outbreak response (Brown et al., 2021), including SARS-CoV-2 genome sequencing to characterize the viruses driving the outbreak and to support contact tracing efforts. Here, we describe the genomic epidemiology of this outbreak, including evidence for frequent SARS-CoV-2 Delta transmission from and between fully vaccinated individuals.

## Results

### A large outbreak of Delta in a highly vaccinated population

**Figure 1:**
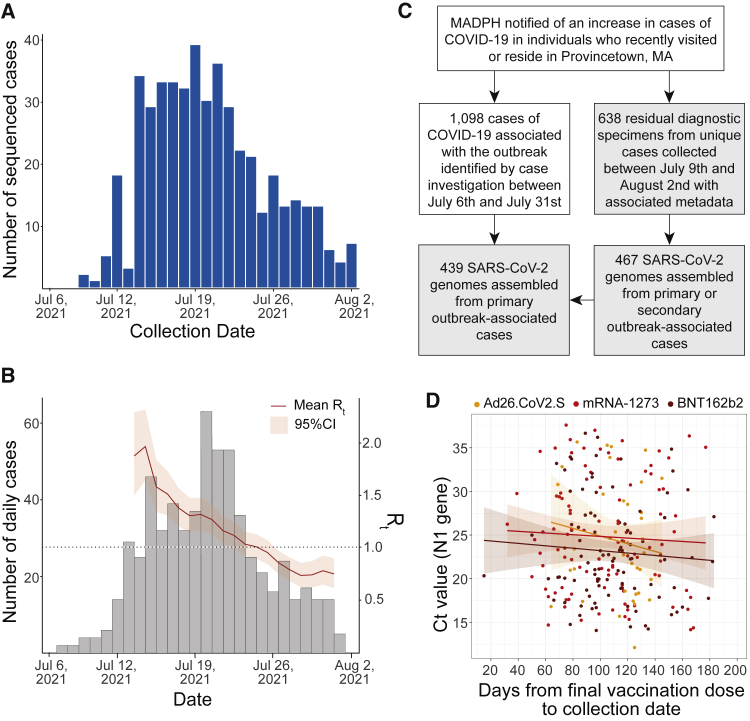
Epidemiology of the COVID-19 Provincetown outbreak and overview of the dataset (A) SARS-CoV-2 genomes in this dataset by collection date. (B) Distribution of all cases in Barnstable County (gray bars) and estimate of reproductive number (*R*_*t*_) (red line) over the course of the outbreak. (C) Flow diagram of sample sets presented here. Gray shading indicates sample sets reported and analyzed in this study. (D) Cycle threshold (Ct) value of the N1 gene for the 313 individuals known to be fully vaccinated by BNT162b2, mRNA-1273, or AD26.CoV2.S; includes linear regression with 95% confidence interval. All Ct values were collected using the same platform and assay. See also Figure S1.

We sequenced SARS-CoV-2 genomes from residual diagnostic specimens collected between July 9th and August 2nd in the Provincetown area as part of outbreak-associated and enhanced community surveillance testing (STAR Methods). We produced high-quality SARS-CoV-2 genomes (unambiguous length ≥24,000 nt and successful gene annotation) from 467 unique individuals. Genomes were well distributed over the time period of the outbreak (Figure 1A), and their numbers closely mirrored the epidemic curve (Brown et al., 2021; Gharpure et al., 2022), with the densest coverage occurring early on when the outbreak was still growing (reproductive number (R_t_) > 1) (Figure 1B). Of these 467 individuals, 439 were primary outbreak-associated cases, representing 40% of the 1,098 known primary outbreak cases (Figure 1C). The remaining cases were secondary cases that had an epidemiologically confirmed link to a primary case. Of the 467 individuals, 346 were MA residents, and the remaining 121 were out-of-state residents.

**Figure S1:**
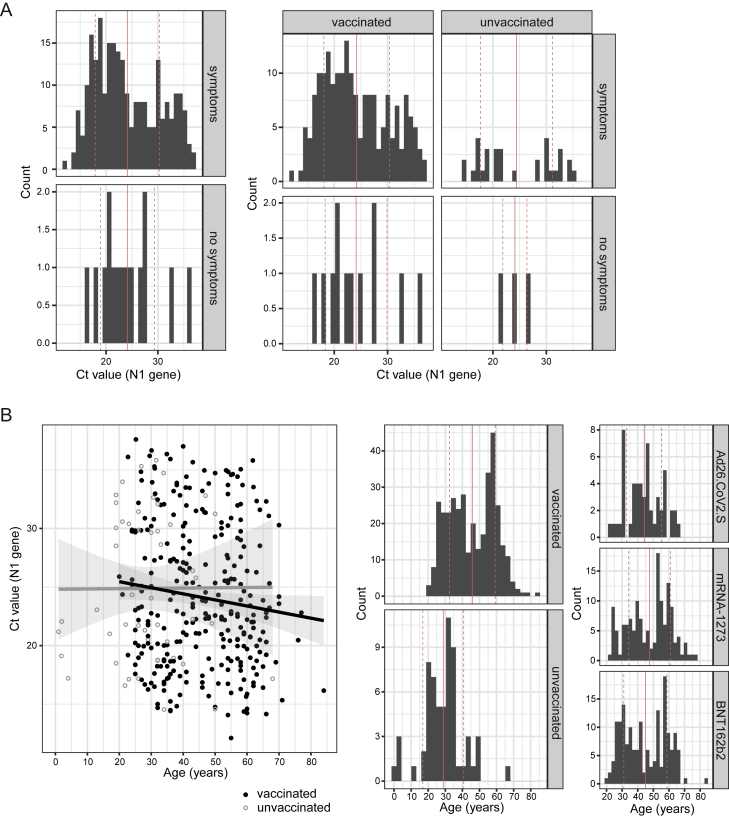
Symptoms, Ct, age, and vaccination status, related to Figure 1 and STAR Methods (A) Ct values in outbreak-associated cases, including cases without genomes (465 individuals passing Ct thresholds). In individuals with multiple samples, the earliest collected sample was used. The presence or absence of symptoms was known for 263 individuals; of these, vaccination status was known for 251. Partially vaccinated individuals were excluded from the analysis at right. In each distribution, the mean is shown by a red line; the mean ± one standard deviation is shown by dashed red lines. All Ct values were collected using the same platform and assay. (B) Ct values by age and vaccination status, and age distributions by vaccination status and vaccine brand in 465 outbreak-associated cases passing Ct thresholds. In individuals with multiple samples, the earliest collected sample was used. Individuals with unknown vaccination status and partially vaccinated individuals are excluded from all six panels. Vaccination status was known for 355 individuals; of these, all had a known age and 290 were known to be fully vaccinated by one of BNT162b2, mRNA-1273, or Ad26.CoV2.S. In each distribution, the mean is shown by a red line; the mean ± one standard deviation is shown by dashed red lines. Scatterplot includes linear regression with 95% confidence interval.

The individuals providing specimens for sequencing were predominantly male (80%), with a median age of 43 years, and 84% were vaccinated, broadly consistent with the outbreak as a whole (Brown et al., 2021; Gharpure et al., 2022). Among the vaccinated individuals, 48% (203) received BNT162b2 (Pfizer-BioNTech), 37% (155) received mRNA-1273 (Moderna), and 14% (58) received Ad26.CoV2.S (Janssen) vaccine products. Consistent with previous reports (Chia et al., 2021; Riemersma et al., 2021), diagnostic cycle threshold (Ct) values, an approximation of viral load, were similar between vaccinated and unvaccinated individuals and between symptomatic and asymptomatic individuals, although there were few of the latter because testing focused on symptomatic cases (Figure S1A). The average time since completion of the primary vaccination series was 111 days. Ct values decreased slightly with increasing time since vaccination, but the trend was not statistically significant (Figure 1D), in line with observations from other cohorts (Singanayagam et al., 2021). Ct values also decreased slightly with increasing age in vaccinated individuals, although not significantly. Unvaccinated individuals were, on average, younger than vaccinated individuals (Figure S1B).

**Figure 2:**
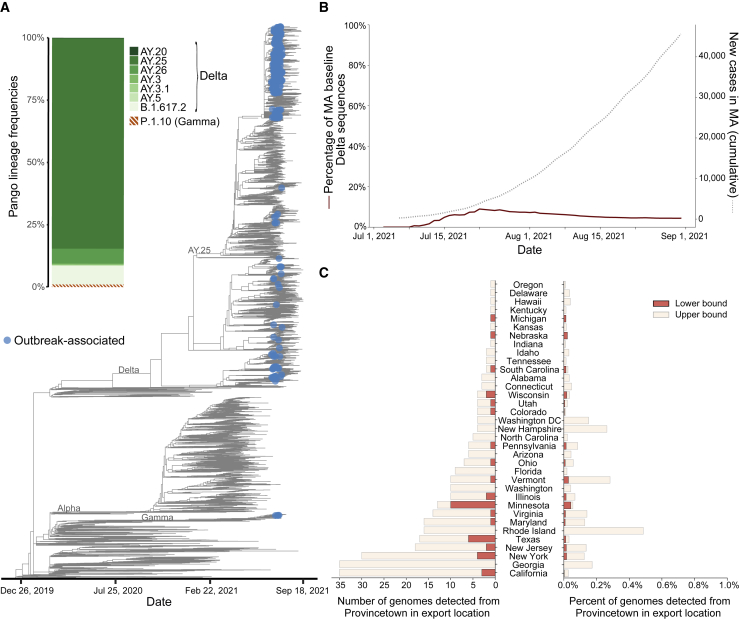
Genomic epidemiology and limited onward impact of the Provincetown outbreak (A) Time tree of SARS-CoV-2 outbreak-associated genomes (blue dots) in the global context. Inset, frequencies of Pango lineages among outbreak-associated genomes. An interactive version of this tree is available at: https://auspice.broadinstitute.org/sars-cov-2/ma-delta/20211005/cluster-unique-usher. (B) Percentage of all Delta-lineage genomes from MA detected by baseline genomic surveillance with the mutational signature of the dominant outbreak cluster (red line). Percentages are calculated and shown per day based on sample collection date. Dashed line: cumulative new cases in MA over the same period (available at: https://www.mass.gov/info-details/covid-19-response-reporting). (C) Numbers of genomes (left) and percentage of Delta-lineage genomes (right) per state that are estimated to descend from the largest cluster in the Provincetown outbreak. Barplots show lower-bound (solid) and upper-bound (faded) estimates, calculated as described in STAR Methods. Note that the scale of the x axis in the plot on the right is in percentages, with a maximum of 1%. See also Figures S2 and S4; Table S1.

**Figure S2:**
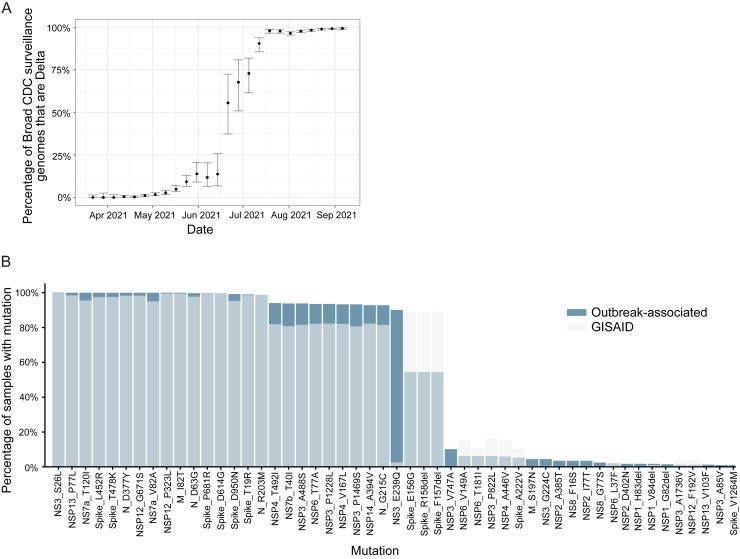
Genomic analysis of Delta lineages and mutations in Massachusetts, related to Figure 2 (A) The proportion by epidemiological week of Delta-lineage sequences among all publicly available baseline surveillance data from MA. Data shown are only those generated by the Clinical Research Sequencing Platform and Viral Genomics Group at the Broad Institute. Error bars denote 95% confidence intervals. (B) The frequency of the 50 most common consensus-level mutations among all outbreak-associated genomes (blue) compared with the proportion of Delta genomes in GISAID with the same mutation (gray). All AY.25 genomes had an amino acid change at position E239Q in ORF3a; however, although rare among publicly available Delta genomes, E239Q is shared across the AY.25 lineage and is of no known functional significance.

Genome sequencing confirmed that the Provincetown outbreak was driven by the Delta lineage: 99% (462/467) of the genomes were Delta, with the remainder being Gamma (Figure 2A). Of the Delta genomes, 84% (394/467) were from the lineage AY.25, a lineage then circulating throughout the US but rarely observed outside North America. The outbreak occurred during a broader rise of the Delta lineage in MA: first detected in MA on March 28th, Delta’s prevalence increased sharply in the weeks preceding the July 4th weekend, from 16% of sequenced SARS-CoV-2 genomes on June 15th to 77% at the start of our sampling period on July 9th, reaching 97% by the end of our sampling period on August 3rd (Figure S2A).

**Figure S3:**
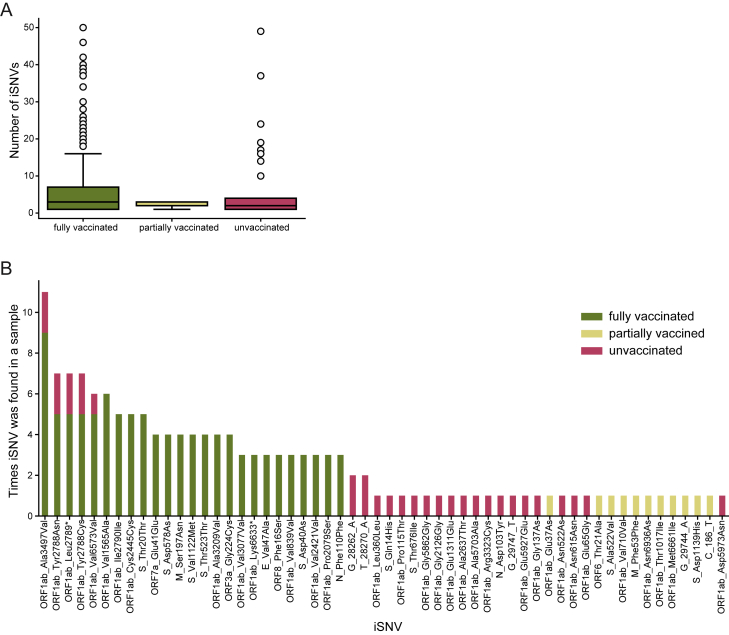
Within-host variation in outbreak-associated samples, related to STAR Methods (A) Total number of iSNVs per individual grouped by vaccination status. (B) The number of observations of each iSNV across all samples. iSNVs are labeled by their gene and amino acid change (if nonsynonymous) or nucleotide position (if synonymous). Bars are colored by the vaccination status of each individual in which a mutation was observed.

We found no evidence of genetic differences of known functional consequence between outbreak-associated and other publicly available Delta genomes or between outbreak-associated genomes from vaccinated and unvaccinated individuals. The Delta genomes in this dataset did not have novel consensus-level variants in the spike protein, nor did they have an increased frequency of any amino acid change of known or suspected functional impact (Figure S2B) (Harvey et al., 2021; Li et al., 2020). While there was little overlap in the intrahost single-nucleotide variants (iSNVs) identified in the vaccinated and unvaccinated individuals, the number of iSNVs was not significantly different between the groups, suggesting little difference in viral genetic diversity with vaccination (Figures S3A and S3B).

### One of many Delta introductions dominated the outbreak

To characterize the transmission dynamics of the Provincetown outbreak, we constructed a maximum-likelihood phylogenetic tree of outbreak-associated SARS-CoV-2 genomes. We included 6,372 additional SARS-CoV-2 genomes sub-selected from publicly available data, enriched for genomes that were genetically, geographically, or temporally relevant (STAR Methods), to enable inference of introductions into and onward spread from the outbreak. Based on this tree, we estimated the number of introductions into Provincetown during the outbreak period, counting a branch as an introduction when an ancestral node on the branch was inferred not to be a part of the outbreak with confidence ≥80%. Using this method, we identified ≥40 distinct introductions, suggesting that the Delta variant was introduced into this population from many sources.

**Figure S4:**
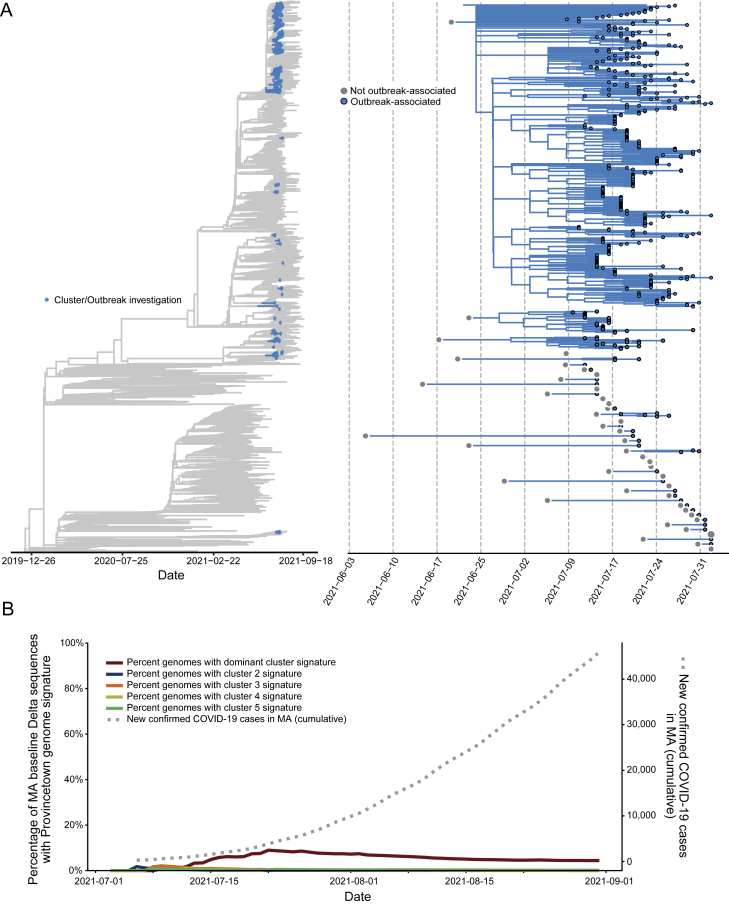
Outbreak introductions and onward spread, related to Figure 2 (A) Left: time tree of outbreak-associated SARS-CoV-2 genomes in a global context colored by association with the Provincetown outbreak (as in Figure 2A). Right: each introduction into Provincetown as inferred from the phylogenetic tree based on a change in ancestral inference of a branch to “outbreak-associated.” Gray dots represent the most recent common ancestor of the clade that was inferred to be from outside of Provincetown. All outbreak-associated samples downstream of each node are shown in the full phylogenetic tree. (B) The percentage of all Delta-lineage baseline surveillance genomes from MA with the mutational signature of the five Delta-lineage clusters among outbreak-associated cases. Three mutations (C8752T, C20451T, and A26759G) are shared by the majority of all outbreak-associated genomes, referred to as the dominant cluster. The remaining four clusters were defined by their characteristic mutations, with cluster 2 defined by G4124A and A5608G; cluster 3 by T7858C and A29257C; cluster 4 by T23131C; and cluster 5 by A26759G, C20451T, C7600T, and T27940C. Percentages per day based on sample collection date and new cumulative cases in MA were plotted over time from July 3rd to August 31st, 2021.

Six of these ≥40 introductions led to clusters (three or more cases) of varying sizes, of which one cluster—comprising 83% (387/467) of outbreak-associated genomes—dominated the outbreak (Figure 2A). The remaining five clusters each accounted for <4% of primary outbreak-associated cases, and all other introductions were associated with a single case each (Figures 2A and S4A). The dominant cluster is defined by three nucleotide mutations, C8752T (ORF1ab N2829K), C20451T (ORF1ab N6729), and A26759G (M G79), all with no known functional significance. The dominant cluster had an estimated time of most recent common ancestor (tMRCA) of June 18th, 2021 (June 12th–June 24th, 2021). The earliest reported genomes within the dominant cluster were from other US states, suggesting that this lineage likely emerged outside MA and was introduced into MA just prior to amplification by the outbreak.

A striking feature of the dominant cluster was the presence of 158 identical consensus genomes (41% of outbreak-associated genomes in the cluster) at the root of the cluster. This pattern—many identical viral genomes within a short time—usually indicates rapid spread from a single individual and is, therefore, a signature of superspreading. In context, this finding suggests that the majority of individuals in this outbreak were infected in Provincetown itself. The public health investigation of the outbreak, however, revealed no evidence that a single exposure site was widely shared among cases. Instead, the genomic and epidemiological data taken together suggest that superspreading of the same viral sequence occurred at multiple locations. This is consistent with several scenarios, including one individual infecting others at multiple locations or several individuals with the same virus, from either a common source or serial infection, transmitting independently. While the initial introduction and early transmission pattern cannot be precisely resolved, the shape of the phylogeny suggests that overdispersion, in which a few individuals are responsible for most transmission events (Endo et al., 2020), continues to play an important role in the COVID-19 pandemic in a landscape dominated by more transmissible variants (Earnest et al., 2021).

### Limited spread beyond Provincetown

We investigated the extent to which cases descending from the outbreak contributed to the subsequent increase in cases, largely driven by the Delta variant, in MA and across the US. Approximately half of the >1,000 outbreak-associated individuals reported residency in MA, with the remainder visiting from 20 other US states (Gharpure et al., 2022), raising the possibility that the outbreak led to widely dispersed secondary transmission. We first looked for the three-mutation genomic signature of the dominant outbreak cluster in viral genomes collected after the outbreak, as part of ongoing state-wide SARS-CoV-2 genomic surveillance in MA (STAR Methods). We found that this signature accounted for a modest and decreasing fraction of Delta infections sequenced in MA over the following weeks, peaking at 9% of all Delta genomes on July 16th and declining to 4% by August 30th (Figure 2B). The smaller outbreak clusters also had a negligible impact on onward spread within the state (Figure S4B).

To quantify the impact of the outbreak more widely in the US, we estimated upper and lower bounds on the spread of the dominant cluster by searching for its descendants in national surveillance data. We estimated an upper bound by identifying all non-MA genomes descended from branches of the dominant cluster with an inferred ancestral origin in MA. Because these genomes could include viruses circulating independently of the Provincetown outbreak, we also estimated a lower bound based on one sub-lineage of the dominant cluster that likely emerged during the outbreak. This sub-lineage was defined by a mutation (T4959C) that first appeared as an iSNV in two individuals from the outbreak (at 39% and 41% frequencies) and had a tMRCA of July 2nd, 2021 (June 24th–July 7th, 2021), near the start of the outbreak, and was not detected outside of MA until July 13th; we assume, therefore, that all members of the sub-lineage derived from the outbreak. Based on these two approaches, we infer that the outbreak led to between 44 and 328 sequenced cases in surveillance data from between 18 and 37 states, collected during the period between July 10th and September 13th, 2021 (Figure 2C). Among the 37 states, New York, California, and Georgia contained the largest number of descendant cases, each representing approximately 10% of the 328 sequenced cases. The upper bound of 328 sequenced cases represents less than 0.08% of the >400,000 Delta genomes sequenced in the same period. Additionally, the outbreak only comprised between 0.01% and 0.048% of sequenced Delta genomes in each exported state (Figure 2C). These findings suggest that, while the outbreak led to some onward transmission, it made at most a modest contribution to later Delta cases within MA and a minimal contribution to cases elsewhere in the US.

### High-confidence transmission from and between vaccinated individuals

**Figure 3:**
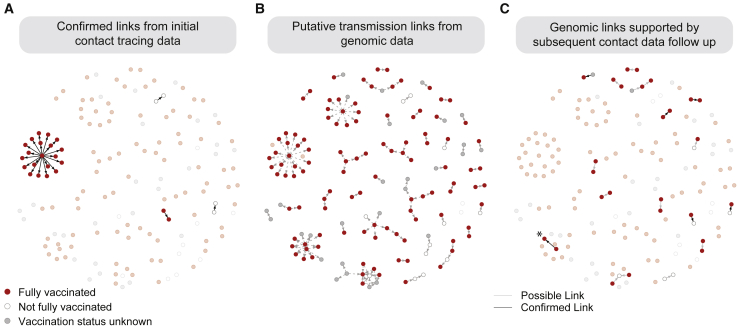
Identification of putative transmission events, including from and between vaccinated individuals (A) High-confidence transmission links (among the 467 cases with genomes) from contact tracing investigations alone, prior to the incorporation of genomic data. (B) Predicted transmission links based on genome sequence, intrahost variants, and symptom onset date. Three transmission links identified by contact tracing but not strongly supported by genomic predictions are shown as dots without connecting lines (see Figure S5A for an example). (C) Genomics-predicted transmission links (from B) further corroborated by additional epidemiological follow-up. Transmission links are indicated as confirmed known links (black lines) or possible links (gray lines) depending on whether contact was confirmed (e.g., members of the same household) or likely (e.g., lived in the same building but not the same home). A confirmed transmission pair that is part of a larger cluster of putative links, described in the text, is marked with an asterisk. In all panels, circles, representing unique individuals, are colored by the vaccination status of the individual at the date of sample collection. See also Figure S5.

**Figure S5:**
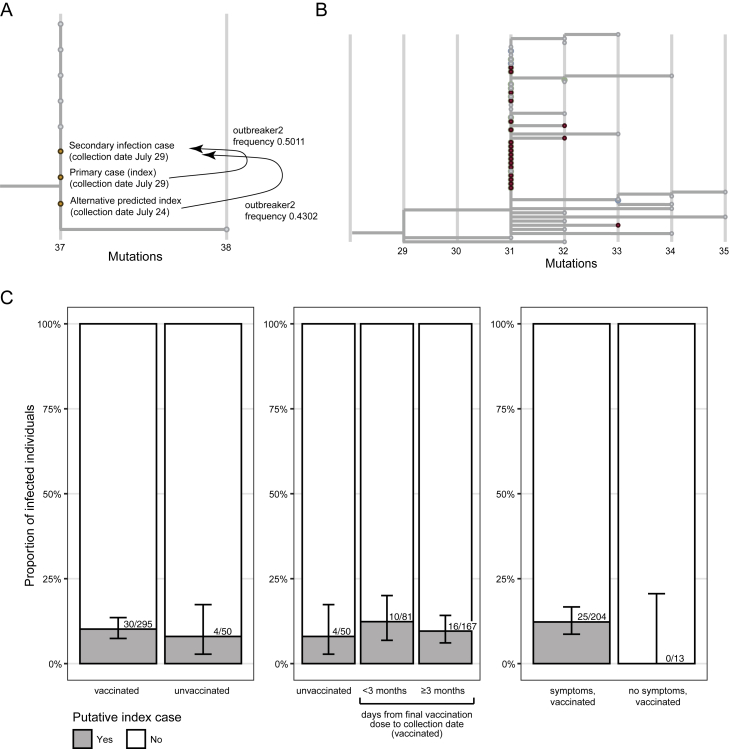
Genomic support for transmission links and transmission predictions by vaccination status and symptoms, related to Figure 3 (A) Maximum-likelihood phylogenetic tree of the only high-confidence transmission pair from contact tracing without strong statistical support in outbreaker2 transmission reconstruction. This pair was in a cluster of six identical consensus genomes with very similar collection dates. No symptom onset date was known for either of the individuals in the pair. Even when incorporating contact tracing information into the model, another sample was predicted as almost equally likely to have been the ancestor of this case. (B) Maximum-likelihood phylogeny of the large cluster of cases associated with a single index case in a close-contact setting. Cases from this epidemiological cluster are colored in dark red. (A) and (B) are part of a larger phylogenetic tree available at https://auspice.broadinstitute.org/sars-cov-2/ma-delta/20211005/cluster-unique-usher. (C) Gray bars, fractions, and 90% confidence intervals indicate the proportion of individuals that were the origin of at least one transmission event predicted by outbreaker2 with a probability of >70%. Individuals are separated by vaccination status (left), days from final vaccination date to collection date (middle), or presence or absence of symptoms in vaccinated individuals (right). 90% binomial confidence intervals were calculated using the exact method through the binom package in R. Using simulations incorporating outbreaker2’s confidence in putative transmission links, we calculate that an infected unvaccinated individual was 0.18–2.11 times as likely to transmit as an infected vaccinated individual. Among fully vaccinated individuals, an infected asymptomatic individual was 0–0.99 times as likely to transmit as an infected symptomatic individual. Our estimates of relative risk are predicated on outbreaker2 correctly estimating the probability that it has chosen the correct index case of each putative transmission.

We next investigated the contribution of vaccinated individuals to transmission in the outbreak, using both public health contact tracing and viral genomic data. Viral genomic data can add resolution to contact tracing by providing an orthogonal measure of the connectivity between cases based on the genetic distance between viruses from infected individuals (Wohl et al., 2016). While conventional contact tracing had limited ability to identify direct transmission events in this outbreak, which spanned multiple locations over several days with many possible contacts at each location, extensive efforts did identify 19 transmission links among primary outbreak-associated cases. These 19 links originated from 17 putative index cases, 16 of whom were vaccinated. Additionally, a cluster of 19 other fully vaccinated cases in a close-contact, residential setting, not otherwise associated with the outbreak, were linked to a single putative outbreak-associated index case. Genomic data were available from both the index and recipient cases for 22 of these 38 putative links identified by contact tracing, linked to four index cases (Figure 3A). Based on the phylogeny, the genomic data were consistent in every instance with the transmission events inferred by contact tracing. The phylogeny also supported that all cases in the close-contact cluster stemmed, probably via direct transmission, from a single introduction, consistent with the single index case suggested by contact tracing (Figure S5B) and underscoring the potential for extensive transmission between vaccinated individuals in prolonged close-contact settings.

Given the limitations of conventional contact tracing in this outbreak, we applied two empirical methods to infer transmission links based on genomic data (STAR Methods). Using only consensus genomes and temporal information to reconstruct a transmission tree, we identified an additional 54 well-supported links, of which 38 involved vaccinated index cases (Figure 3B). However, consensus-level genome data were also limited in their ability to infer transmission links, in this case, because the low viral diversity in the outbreak often made it difficult to identify direct connections within a cluster. To address this, we applied a second, complementary method, using sub-consensus genome diversity (iSNVs) to identify putative index cases within clusters. This approach can suggest the direction of transmission when several cases collected close in time have identical consensus genomes; variants typically start as iSNVs and become consensus alleles in secondary cases (McCrone and Lauring, 2018; Sobel Leonard et al., 2017). Using iSNVs, we identified a further 51 putative transmission links, 44 of which have a vaccinated index case (Figure 3B). In total, genomic data suggested 105 transmission links that were not detected through initial contact tracing.

Further epidemiological investigation provided information about 18 of the transmission links identified from genomic data (Figure 3C), including shared household, workplace, or hospitality settings, which supported the genomic inference. All 18 of these epidemiologically supported contacts had been identified by initial contact tracing efforts but were not classified as high-confidence because of the stringency of the epidemiological definition of a high-confidence link. Six of the 18 confirmed links were classified as involving known contacts and 12 as involving plausible contacts (e.g., living in the same building). In four of the six known contact links and nine of the 12 plausible contact links, the putative index case was vaccinated. Most of these predicted links involved a single secondary case; however, one putative index case was linked to nine secondary cases based on genomic data (six of the nine being vaccinated individuals). One of these nine secondary cases was identified as a known contact, the index having reportedly visited the restaurant where the contact was employed (Figure 3C), raising the possibility of more extensive but unknown transmission from this vaccinated individual. Contact tracing could neither confirm nor refute the remaining links identified by genomic inference. This could be due to the complex epidemiology of the outbreak, the inevitable incompleteness of contact tracing data, or limitations of the genomic inference.

Due to the small number of unvaccinated cases in our dataset, we were unable to meaningfully compare rates of secondary transmission by vaccination status; we were also not able to meaningfully compare rates of secondary transmission by time since vaccination (Figure S5C). Our observations are consistent with a recent study from the United Kingdom (Singanayagam et al., 2021), which observed transmission from vaccinated household contacts. We did note that among vaccinated individuals, there were no identified asymptomatic index cases in this analysis, suggesting a lower risk of transmission from asymptomatic individuals, but the large uncertainty on the relative risk (95% confidence interval: 0–99% risk of transmission from an asymptomatic vaccinated versus symptomatic vaccinated individual) and possible biases in this observational dataset make drawing conclusions difficult. However, despite the complexities of the epidemiology, difficulty of contact tracing, and low viral genetic diversity in this outbreak, genomic and epidemiological data combined provide strong support for 22 transmissions from vaccinated individuals and suggestive evidence of a further nine, while genomic data alone provide suggestive evidence for an additional 69. These data suggest that, despite high antibody responses observed for vaccinated individuals from this outbreak following breakthrough infection (Collier et al., 2021), transmission to and from vaccinated individuals is common in some settings.

## Discussion

The outbreak of SARS-CoV-2 in Provincetown during and after the July 4th weekend was the first large outbreak of the Delta variant in a highly vaccinated population in the US. The robust public health response permitted extensive epidemiological and genomic characterization of the outbreak, the structure of transmission within it, and the role of vaccinated individuals, and offers generalizable insights for containing future outbreaks of Delta and other highly transmissible lineages of SARS-CoV-2.

The Provincetown outbreak raised public health concerns and attracted international attention, primarily due to the prevalence of symptomatic breakthrough infections and the potential occurrence of transmission from vaccinated individuals. Consistent with other recent reports (Chia et al., 2021; Riemersma et al., 2021), breakthrough infections with Delta, while often symptomatic and with moderate to high viral loads, were typically mild. Confidently assigning transmission links between individuals was unusually challenging—conventional contact tracing was difficult because of the many locations with dense potential contacts involved, while genomic inference of transmission was hindered by the low overall genetic diversity and large fraction of identical genomes. Nonetheless, using viral genomic data to identify and prioritize plausible connections between cases, followed by more detailed epidemiological investigation, identified several likely instances of transmission between fully vaccinated individuals. Furthermore, genomic data suggested many additional links that were not captured by contact tracing due to the complexity of the outbreak or the conservative epidemiological definition of close contact, providing a richer picture of the underlying dynamics of the transmission in this outbreak and possibly serving as a model for future investigations. To this end, further improvements to transmission inference tools would be helpful in adding additional resolution to densely sampled large outbreaks.

The size of the Provincetown outbreak—over one thousand cases—and its rapid early growth demonstrate that in densely crowded events and indoor conditions, the SARS-CoV-2 Delta variant can cause a large outbreak even in a highly vaccinated population. However, the Provincetown outbreak did not contribute substantially to the increase in Delta cases in MA or elsewhere in the US. The high rates of vaccination and the swift public health response (Brown et al., 2021), which included deployment of mobile testing, a local indoor masking mandate, and an extensive outreach campaign, likely contributed to the short duration and restricted impact of the outbreak. Additionally, the active engagement of the affected community in the epidemiological response, possibly influenced by historical public health outreach in the gay community, may have helped mitigate the impact of the outbreak (Marks, 2021). The rapid decline and limited impact of the outbreak suggest that while Delta-driven outbreaks are not eliminated by high vaccination rates, they can be controlled with well-understood public health measures.

### Limitations of the study

The dataset used in this study consisted of samples from individuals who chose to be tested for SARS-CoV-2 during and after the outbreak; it is possible that willingness to be tested was not uniform with respect to the categories considered in the study, in particular with respect to vaccination status. More generally, the outbreak was incompletely sampled, and the viral population involved had low genetic diversity, which meant that our ability to comprehensively infer individual transmission links was limited. We had limited epidemiological data, including vaccination status, from cases with residence outside of MA, which reduced our power to identify transmission links from and between vaccinated individuals. Inferences about sources of introductions into and onward transmission from the outbreak were limited by local testing and sequencing rates, which vary substantially across the US.

## STAR★Methods

### Key resources table

**Table:** 

REAGENT or RESOURCE	SOURCE	IDENTIFIER
**Biological sample**		

Anterior Nares swabs	Massachusetts Department of Public Health & regional testing facilities	N/A

**Critical commercial assays**		

Thermo Fisher MagMAX Viral RNA Isolation kit	Thermo Fisher	Cat #AM1939
TaqPath,1Step RTqPCR MtrMix	Thermo Fisher	Cat #A15300
CDC 2019-Novel Coronavirus (2019-nCoV) Real-Time RT-PCR Diagnostic Panel	CDC	Cat #2019-nCoVEUA-01
NEBNext ARTIC SARS-CoV-2 FS Library Prep Kit	New England BioLabs	Cat #E7658L
NovaSeq 6000 SP Reagent Kit v1.5 (300 cycles)	Illumina	Cat #20028400
NovaSeq XP 2-Lane Kit v1.5	Illumina	Cat #20043130
MagNA Pure LC Total Nucleic Acid Isolation Kit	Roche	Cat #03038505001
SuperScript IV reverse transcriptase	Thermo Fisher Scientific	Cat #18090200
Random Primer Mix	New England BioLabs	Cat #S1330S
AMPure XP	Beckman Coulter	Cat #A63881
Q5 Hot Start High-Fidelity 2X Master Mix	New England BioLabs	Cat #M0494S
Illumina DNA Prep	Illumina	Cat #20018705
IDT for Illumina DNA/RNA UD Indexes Set A-D	Illumina	Cat #20027213-16
MiSeq V2 300 cycle kit	Illumina	Cat #MS-102-2002

**Deposited data**		

RNA sequencing data	NCBI SRA	BioProject: PRJNA715749, PRJNA686883
GISAID consensus genomes	GISAID	See Table S1
GenBank consensus genomes	NCBI GenBank	BioProject: PRJNA715749, PRJNA686883

**Oligonucleotides**		

ARTIC Network V3 SARS-CoV-2 primers	ARTIC Network	https://github.com/artic-network/artic-ncov2019/tree/master/primer_schemes/nCoV-2019/V3

**Software and algorithms**		

LoFreq version 2.1.5	Wilm et al., 2012	https://csb5.github.io/lofreq/
viral-ngs 2.1.28	Broad Institute	https://dockstore.org/workflows/github.com/broadinstitute/viral-pipelines/sarscov2_illumina_full:master
Nextstrain v3.0.3	Hadfield et al., 2018	https://dockstore.org/workflows/github.com/broadinstitute/viral-pipelines/sarscov2_nextstrain_aligned_input:master
FastTree version 2.1.11	Price et al., 2009 and 2010	http://www.microbesonline.org/fasttree/
baltic	GitHub	https://github.com/evogytis/baltic
EpiEstim v4.0.1	Cori et al., 2013	https://cran.r-project.org/web/packages/EpiEstim/index.html
outbreaker2 (version 1.1.2)	Campbell et al., 2018	https://cran.r-project.org/web/packages/outbreaker2/index.html
Nextclade CLI version 1.3.0	Aksamentov et al., 2021	https://github.com/nextstrain/nextclade

### Resource availability

#### Lead contact

Further information and requests for resources and reagents should be directed to and will be fulfilled by the lead contact, Katherine J. Siddle (kjsiddle@broadinstitute.org).

#### Materials availability

This study did not generate new unique reagents.

### Experimental model and subject details

#### Ethical approvals

The research project (Protocol #1603078) was reviewed and approved by the Massachusetts Department of Public Health (MADPH) Institutional Review Board (IRB) and covered by a reliance agreement at the Broad Institute. Residual diagnostic specimens were sequenced under a waiver of consent from the MADPH IRB. An additional non-human subjects research and an exempt determination (EX-7080) were made by the Harvard Longwood Campus Institutional Review Board and the Broad Institute Office of Research Subject Protections, respectively, for the analysis of de-identified aggregate and publicly available data. Some study staff maintain dual affiliations with Mass General Brigham and the Broad Institute, but this research was conducted solely at the Broad Institute, Harvard University, and MADPH.

### Method details

#### Sample collection and case definitions

State and local authorities identified cases linked to the Provincetown outbreak using travel history and exposure data from the state COVID-19 surveillance system and follow-up case investigation. A primary outbreak-associated case was defined as receipt of a positive SARS-CoV-2 test result (nucleic acid amplification test or antigen test) ≤14 days after travel to or residence in Provincetown between July 3rd and July 17th, 2021. The majority of specimens in the present dataset were collected through mobile testing deployed in Provincetown by the MADPH following identification of the outbreak (Brown et al., 2021). Cases analyzed in this study ranged in age from 0 to 105 years old; 125 were female and 513 were male. Among cases with genome sequencing information, the age range was the same as for all patients and included 94 females and 373 males.

Cases that did not meet the above criteria as primary cases but were collected in Provincetown or had an epidemiologically confirmed link to a primary case (i.e., secondary cases) are collectively referred to here as “outbreak-associated.” COVID-19 vaccine breakthrough cases were defined on the basis of either i) documentation from the state immunization registry of completion of COVID-19 vaccination as recommended by the CDC Advisory Committee on Immunization Practices ≥14 days before specimen collection or ii) self-reported vaccination dose(s) indicating completion of COVID-19 vaccination ≥14 days before sample collection during follow-up case investigations. Individuals who had received at least one vaccine dose ≥1 day before sample collection but did not meet these criteria were defined as partially vaccinated.

#### SARS-CoV-2 detection and sequencing

We identified specimens for viral genome sequencing linked to the Provincetown outbreak based on the above criteria and which were submitted to either the Massachusetts State Public Health Laboratory (MASPHL) or the Broad Institute for testing, following confirmation by diagnostic RT-qPCR test.

For specimens submitted to the Broad Institute, total RNA was extracted from inactivated Anterior Nares (AN) swabs using the Thermo Fisher MagMAX Viral RNA Isolation kit and presence of virus was determined by an RT-qPCR assay detecting the N1 and N2 gene regions of the virus used under Emergency Use Authorization at the Broad Institute Clinical Research Sequencing Platform in a CLIA-compliant diagnostic laboratory. Positive controls across all RT-qPCR plates containing samples analyzed as part of this dataset were highly consistent (N1 gene mean Ct = 26.61, standard deviation = 0.59; N2 gene mean Ct = 28.01, standard deviation = 0.59), demonstrating minimal variation between batches. Ct values for the N1 gene were used to compare viral titers between individuals; samples for which the positive control target (human ribonuclease P gene) had a Ct>32 were excluded from analyses involving Ct values to avoid biasing towards high viral loads. Specimens with a positive (N1 and N2 detected) or inconclusive (only one of N1 or N2 detected) test result, regardless of diagnostic Ct value, were re-extracted from the source material. Illumina sequencing libraries were prepared using the NEBNext ARTIC v3 SARS-CoV-2 FS Library Prep Kit. Libraries were sequenced on Novaseq SP flowcells with 75-nucleotide paired-end reads. During library preparation, some volumes were adjusted from manufacturer recommendations to accommodate 384-well plate reactions and high-throughput automated processing.

For specimens determined to be positive for SARS-CoV-2 based on RT-qPCR at the MASPHL, total RNA was extracted using the Roche MagNA Pure Total Nucleic Acid Isolation kit. Following extraction, samples proceeded to cDNA synthesis, amplification using ARTIC v3 SARS-CoV-2 PCR primers, and library preparation with the Illumina DNA Prep kit including bead-based normalization of library concentration prior to library pooling. Libraries were sequenced on Illumina MiSeq with 2x150 paired-end reads.

#### SARS-CoV-2 genome assembly and analysis

For genomes generated at the Broad Institute, we conducted all analyses using viral-ngs 2.1.28 on the Terra platform (app.terra.bio). All of the workflows named below are publicly available via the Dockstore Tool Registry Service (dockstore.org/organizations/BroadInstitute/collections/pgs). Briefly, samples were demultiplexed, reads were filtered for known sequencing contaminants, and SARS-CoV-2 reads were assembled using a reference-based assembly approach with the SARS-CoV-2 isolate Wuhan-Hu-1 reference genome GenBank: NC_045512.2 (*sarscov2_illumina_full.wdl*). For genomes generated at the MASPHL, all analyses were executed on a local, on-premise, Linux compute machine at the MASPHL. We processed all raw read data using a reference-based consensus calling method with the same NC_045512.2 reference genome. The workflow is publicly available on GitHub (github.com/AndrewLangvt/genomic_analyses/blob/main/workflows/wf_viral_refbased_assembly.wdl). On both Broad Institute and MASPHL alignments, we used LoFreq version 2.1.5 to call intrahost single nucleotide variants (iSNVs) with default parameters (minimum read depth ≥10, strand bias <85%, and default iSNV quality scoring) (Wilm et al., 2012). Variants with frequency ≥3% and in positions with read depth ≥200 were used for downstream analysis.

Assembled genomes meeting the CDC criteria for submission to public repositories (unambiguous length ≥24,000 nt and successful gene annotation) were deposited in NCBI GenBank and GISAID (Elbe and Buckland-Merrett, 2017) immediately upon completion. Raw reads for all samples (including those that did not produce a successful genome) were deposited in NCBI SRA. All NCBI data were deposited under BioProject: PRJNA715749 (Broad) or BioProject: PRJNA686883 (MASPHL) and have been tagged with the BioSample attribute purpose_of_sequencing set to a value of “Cluster/Outbreak investigation” for primary and secondary outbreak-associated cases identified by MADPH epidemiologists or “Targeted surveillance (non-random sampling)” for samples collected as part of enhanced surveillance efforts but where no primary or secondary link to the outbreak was known. In the main text, these groups are together referred to as outbreak-associated, as described in “Sample collection and case definitions.”

Where an individual produced multiple positive tests, we used for analysis the most complete genome that met the CDC criteria for submission to public data repositories; if two or more genomes were of the same length, we selected the genome from the earlier collection time. We confirmed that genomes generated from the same patient were generally concordant; in two cases, genomes obtained from the same individual differed by a single mutation and in one case, a pair of genomes from one individual differed by two mutations. These mutations did not impact phylogenetic assignment or other inferences and likely result from lower coverage for one of the samples.

Coverage of the spike gene was on average 90.11% across assembled genomes meeting the CDC criteria for submission to public repositories. Regions of missing coverage were consistent with ARTIC V3 primer regions reported to be absent in Delta genomes owing to mutations in the primer binding sites. To prevent these gaps from impacting interpretation of the data, these regions were masked from phylogenetic analysis (see below).

#### Phylogenetic Tree construction

We constructed a maximum-likelihood (ML) phylogenetic tree (Sagulenko et al., 2018) with associated visualizations using a SARS-CoV-2-tailored Augur pipeline (Huddleston et al., 2021) (*sarscov2_nextstrain_aligned_input*), part of the Nextstrain project (Hadfield et al., 2018), adapted from github.com/nextstrain/ncov, with the entirety of ARTICv3 amplicons 64, 72, and 73 (Delta dropout regions) masked from tree construction.

We included contextual genomes from the GenBank database (downloaded October 1st, 2021) using two subsampling schemes to prioritize genomes genetically, geographically, and temporally close to outbreak-associated genomes. First, we used a focal weighted subsampling scheme (using the Nextstrain script priorities.py) to prioritize genomes genetically, geographically, and temporally close to our outbreak-associated genomes (https://github.com/broadinstitute/nextstrain-builds/blob/main/builds/broad-usa-builds.yaml#L125). Second, we forced inclusion of a set of contextual genomes identified by phylogenetic proximity to the outbreak genomes by concatenating: a) a list of samples obtained by performing a sequence search in UCSC UShER (Turakhia et al., 2021) against the "GISAID, GenBank, COG and CNCB (3,960,091 genomes)" database on September 28th, 2021, with "Number of samples per subtree showing sample placement" set to 100, resulting in the identification of 3,970 proximal samples; and b) a list of samples obtained by constructing a phylogenetic tree using FastTree (Price et al., 2009 and 2010) (version 2.1.11) on a multiple sequence alignment masked as described above (retrieved from GISAID August 20th, 2021) of a random sample of 194,716 Delta lineage viral sequences, using iterative tree refinement followed by a greedy depth-first search to identify outbreak-enriched clades (clades with >10% of total leaf nodes being outbreak samples). Concatenation of the above lists and outbreak-associated genomes followed by deduplication resulted in forced inclusion of 6,372 genomes.

We used the contextualized ML phylogeny to estimate the number of introductions that seeded the Provincetown outbreak and the number of exports descending from the outbreak. To do so, we assigned a binary trait to each genome in the phylogeny, outbreak-associated or not outbreak-associated, and used Nextstrain’s ancestral inference (Hadfield et al., 2018) to infer the state of that trait for each internal node in the tree. We then defined an introduction as a trait change in a tree branch from not outbreak-associated to outbreak-associated. More specifically, we used baltic (https://github.com/evogytis/baltic) to extract from the phylogeny all changes in state of internal nodes from not outbreak-associated to outbreak-associated with an inferred date of June 15th or later. We define an introduction when an ancestral node on the branch was inferred to be not outbreak-associated with confidence ≥80%. Each of these introductions, with the resulting cluster, was extracted from the ML tree and visualized using matplotlib.

#### Persistence and export of outbreak-associated mutations

We inferred the geographic ancestral origin of each internal node at the level of division (e.g., US State). We defined exports as changes in inferred geographic division starting from a branch inferred to be from MA. To find exports from the dominant cluster of the Provincetown outbreak, we traversed the tree from outbreak-associated tips to the earliest internal node with an inferred date on or after July 3rd. From that collection of nodes we traversed the tree towards later time points. We defined the upper bound on downstream exported transmissions as the number of nodes outside MA, and counted the number of descendant genomes outside MA.

To quantify the impact of the Provincetown outbreak on subsequent spread of Delta lineage viruses in MA and to identify novel or frequent mutations of functional consequence, we compared the frequency of mutations detected in outbreak-associated genomes to their frequency in publicly available data. We downloaded from GISAID (Elbe and Buckland-Merrett, 2017) all MA Delta genomes with collection date between July 3rd and August 30th, 2021, and purpose of sequencing listed as “baseline surveillance.” All sequences were processed with Nextclade CLI version 1.3.0 (Aksamentov et al., 2021) and custom python code. We used nucleotide substitutions that define each of the five Delta clusters to count the number of publicly available sequences in each cluster and used the sample collection date to calculate a daily frequency of each cluster compared to all baseline surveillance Delta genomes.

#### Estimate of effective reproductive number

We used a model based on the parametric_si method in the R package EpiEstim v4.0.1 (Cori et al., 2013) to estimate the effective reproductive number (*R*_*t*_), the average number of secondary cases per infectious case at a given time, for the Provincetown outbreak using case counts of outbreak-associated MA cases with specimen collection dates from July 6th through July 31st, 2021. Our estimates assume that Delta has a serial interval of 2.3 days with a standard deviation of three days (Zhang et al., 2021); that the serial interval is the same for vaccinated and unvaccinated individuals; and that there are no negative serial intervals, where a contact becomes symptomatic before the index. *R*_*t*_ is calculated using $R_{t}=\frac{c\left(t\right)}{c\left(t-\tau \right)}$, where *c*(*t*) is the incidence at time and is the mean value of the serial interval.

#### Estimation of transmission events

We used the R package outbreaker2 (version 1.1.2) (Campbell et al., 2018) to estimate the probability of direct transmission between each pair of individuals based on consensus genome sequences. Where symptom onset date was not available, we used the sample collection date. We supplied a Gamma distribution based on a previously described mean and standard deviation of the Delta (B.1.617.2)-specific generation interval and incubation period (Zhang et al., 2021). For the genomes where contact tracing information was available, we included a matrix of contact tracing data. We left all other input parameters as unknowns to be estimated by outbreaker2 and allowed for transmission events from individuals not included in the dataset. We ran the outbreaker2 Markov chain Monte Carlo (MCMC) algorithm six times with 1,000,000 iterations each, discarding the first 10% as burn-in. After averaging the transmission probabilities across the six runs, we defined well-supported transmission events as those with mean probability >70%. We selected this method over other available tools due to its ability to incorporate both genomic and contact-tracing data into transmission predictions.

For the iSNV analysis of transmission links, we conservatively identified a putative transmission when 1) the detected iSNV frequency in the contact was ≥50%, 2) the index and contact consensus sequences only differed by one consensus-level change, 3) the contact appeared downstream of the index in a divergence phylogeny, and 4) the symptom onset date of the index was ≥2 days before that of the contact.

#### Estimation of transmission rates

To assess differences in transmission rates between vaccinated and unvaccinated individuals, we counted individuals in the two categories who were index cases in well-supported transmissions and used those counts to calculate the relative risk of transmission. A large cluster of secondary cases associated with a known residential close-contact setting was excluded from the analysis. We estimated the relative risk and calculated confidence intervals by constructing via simulation the likelihood function for the observed number of transmissions from the two categories, based on the number of samples in each category, under a model with one free parameter, the relative risk of transmission. In the model, error in inferring the index case was accommodated by replacing the vaccination status of a fraction of true index cases with the status of a sample drawn randomly from the population; the probability of replacement was itself drawn from the distribution of estimated uncertainties in index case assignment. Four million iterations of the simulation yielded a maximum likelihood estimate of the relative risk and a 95% confidence interval (determined from a decrease in the likelihood of 1.92 logs). A similar procedure was used for symptomatic/asymptomatic transmission from vaccinated individuals, with 100,000 iterations of the simulation.

### Quantification and statistical analysis

All statistical analyses were performed in R (The R Core Team, 2017) or python 3. For comparisons of Ct values or ages by vaccination status, vaccine brand, or presence or absence of symptoms we performed a two-sample Kolmogorov-Smirnov test using the function ks.test implemented in the dgof package. For comparisons of Ct values by age or time since vaccination we generated R^2^ and p-values using the lm and summary functions implemented in the stats package. All additional statistical details including sample numbers can be found in the respective figure legends. For comparisons of the number of iSNVs by vaccination status we performed an independent t-test using the statistics functions from the SciPy package. For estimations of transmission rates all statistical details can be found in the figure legend and the relevant methods detail section. No subsampling or randomization was performed for any statistical test and no samples were excluded except where explicitly stated in the method details.

## Data Availability

• All SARS-CoV-2 genomes, patient metadata, and raw sequencing reads have been deposited to NCBI under BioProject: PRJNA715749 or BioProject: PRJNA686883 in GenBank, BioSample, and SRA databases, respectively. All genomes produced in the present study are also available on GISAID. All data is publicly available as of the date of publication. Accession numbers of additional publicly available data analyzed in this paper are available in Table S1. • All code used for sequence data processing, genome assembly, and phylogenetic analysis is publicly available either via the Dockstore Tool Registry Service (dockstore.org/organizations/BroadInstitute/collections/pgs) or on GitHub (github.com/AndrewLangvt/genomic_analyses/blob/main/workflows/wf_viral_refbased_assembly.wdl). • Any additional information required to reanalyze the data reported in this paper is available from the lead contact upon request.

## Supplemental information

Table S1. Acknowledgement table of GISAID data contributors of genome sequences used for phylogenetic tree reconstruction, related to Figure 2
